# Topography of Cholinergic Nerve Terminal Vulnerability and Balance Self-Efficacy in Parkinson’s Disease

**DOI:** 10.31083/j.jin2309178

**Published:** 2024-09-24

**Authors:** Jaimie Barr, Robert Vangel, Prabesh Kanel, Stiven Roytman, Chatkaew Pongmala, Roger L. Albin, Peter J. H. Scott, Nicolaas I. Bohnen

**Affiliations:** 1 Department of Radiology, University of Michigan, Ann Arbor, MI 48109, USA; 2 Functional Neuroimaging, Cognitive, and Mobility Laboratory, University of Michigan, Ann Arbor, MI 48106, USA; 3 Parkinson’s Foundation Research Center of Excellence, University of Michigan, Ann Arbor, MI 48109, USA; 4 Morris K. Udall Center of Excellence for Parkinson’s Disease Research, University of Michigan, Ann Arbor, MI 48109, USA; 5 Neurology Service and GRECC, VA Ann Arbor Healthcare System (VAAAHS), Ann Arbor, MI 48105, USA; 6 Department of Neurology, University of Michigan, Ann Arbor, MI 48109, USA

**Keywords:** Parkinson’s disease, fear of falling, self-efficacy, positron emission tomography, cholinergic systems

## Abstract

**Background:**

Postural instability and gait disturbances (PIGD) represent a significant cause of disability in Parkinson’s disease (PD). Cholinergic system dysfunction has been implicated in falls in PD. The occurrence of falls typically results in fear of falling (FoF) that in turn may lead to poorer balance self-efficacy. Balance self-efficacy refers to one’s level of confidence in their ability to balance while completing activities of daily living like getting dressed, bathing, and walking. Lower self-efficacy, or greater FoF during these activities is a function of motor, cognitive, and emotional impairments and may impact quality of life in PD. Unlike known cholinergic reduction, especially in the right lateral geniculate and caudate nuclei, little is known about the role of cholinergic transporters in FoF or mobility self-efficacy in PD.

**Methods:**

[^18^F]fluoroethoxybenzovesamicol ([^18^F]FEOBV) positron emission tomography (PET) studies were conducted to assess vesicular acetylcholine transporter (VAChT) expression in 126 patients with PD (male (m) = 95, female (f) = 31). Participants had a mean age of 67.3 years (standard deviation (SD) = 7.1) and median Hoehn Yahr stage of 2.5. Patients also completed the Short Falls Efficacy Scale (sFES-I) as a survey measure of concerns about falling. [^18^F]FEOBV data were processed in Statistical Parametric Mapping (SPM) using a voxel-wise regression model with sFES-I scores as the outcome measure.

**Results:**

Reduced [^18^F]FEOBV binding in tectum, metathalamic (lateral more than medial geniculate nuclei), thalamus proper, bilateral mesiotemporal (hippocampal, parahippocampal, fusiform gyri and fimbriae), and right cerebellar lobule VI significantly associated with higher sFES-I scores (*p <* 0.05, family-wise error (FWE) correction after Threshold-Free Cluster Enhancement (TFCE)).

**Conclusions:**

Unlike the more limited involvement of the brainstem-thalamic complex and caudate nuclei cholinergic topography associated with falls in PD, cholinergic reductions in the extended connectivity between the thalamic complex and the temporal limbic system via the fimbriae associates with FoF. Additional cholinergic changes were seen in the cerebellum. The temporal limbic system plays a role not only in episodic memory but also in spatial navigation, scene and contextual (*e.g.*, emotional) processing. Findings may augur novel therapeutic approaches to treat poor mobility self-efficacy in PD.

**Clinical Trial Registration:**

No: NCT02458430. Registered 18 March, 2015, https://www.clinicaltrials.gov/study/NCT02458430; No: NCT05459753. Registered 01 July, 2022, https://www.clinicaltrials.gov/study/NCT05459753.

## 1. Introduction

Postural instability and gait difficulties (PIGD), such as imbalance, slow walking, freezing of gait, and falls are among the cardinal motor features of Parkinson’s disease (PD). PIGD motor features represent a significant cause of disability and negatively impact the quality of life for people with PD [1]. Among them, falls are especially hazardous to a patient’s health as they may account for a three-fold increase in risk of acute hospitalizations in PD [2]. Dopaminergic medications reliably improve some symptoms like bradykinesia and rigidity but are not consistently efficacious in treating PIGD symptoms [3]. While dopaminergic medications may initially have beneficial effects on gait and balance, these benefits decrease with advancing disease, as symptoms become increasingly resistant to dopaminergic medication [4]. Not only do these symptoms increase the risk to physical health, but PIGD symptoms may also negatively impact mental health.

While PD has long been thought of as predominantly a motor syndrome, it is vital that the psychological features of PD do not go unnoted. Within the study of psychology, self-efficacy refers to an individual’s perception of their ability to successfully complete a task [5]. Lower self-efficacy in people with PD has been associated with lower quality of life, poorer motor symptoms, and greater activity avoidance [6,7]. It has been well documented that people with PD have lower balance self-efficacy, affecting important activities of daily living like walking, dressing, bathing, and climbing stairs [8,9]. One study found that PD patients with higher fear of falling (FoF), demonstrated different gait parameters, including slower gait speed, shorter stride length, and greater stride width compared to patients with lower FoF [10]. Other research on self-perceived balance and falls suggests that lower balance confidence may predict increased fall risk, independent of actual motor impairments. One study showed that FoF was a significant predictor of recurrent fall risk in PD after adjusting for prior fall history and motor symptoms [11]. Self-efficacy represents a potentially modifiable risk factor contributing to falls. A better understanding of the mechanisms underlying self-efficacy may contribute to a better understanding of fall risk in PD and lead to more accurate identification of at-risk patients and the development of prevention strategies.

This may be compounded with other psychological symptoms of PD such as anxiety, depression and catastrophizing. Researches have shown that these psychological factors, particularly catastrophizing, may lead to FoF avoidance behaviors [12,13]. These avoidance behaviors preclude people with PD from walking in crowds, new places, and recreational or leisure activities [14]. Thus, FoF avoidance behaviors may prevent people with PD from taking part in exercise and social gatherings, increasing sedentariness, and thereby frailty, which may further diminish their health and quality of life.

New neurotransmitters and neurological systems need to be explored to better understand and address FoF from a comprehensive neurobiological perspective. In addition to the well documented loss of dopamine in PD, changes in the neurotransmitter acetylcholine (ACh) may also play a large role in the progression of the disease. Within the brain three major sources of cholinergic projections have been identified; the basal forebrain, the pedunculopontine nucleus-laterodorsal tegmental complex (PPN-LDTC), and interneurons of the striatum [15]. Together, these areas innervate a large portion of the brain. Of note, the PPN-LDTC provides cholinergic inputs to the thalamus and cerebellum [16], while the basal forebrain provides inputs to the cortex [17]. Denervation of the striatum is a key factor in the symptomology of PD as the basal ganglia and striatum are vital for motor control. Losses of dopamine activity and availability in combination with changes in cholinergic innervation may further provide a basis for understanding PD symptomology and disease progression. Changes in the activity of ACh have been implicated in decreased mobility in PD, especially increased occurrences of falls [18]. Furthermore, ACh plays an important role in executive function and attention, both of which play a vital role in safe mobilization [19].

Although it is clear that ACh plays a role in cognition and motor functions, the role of ACh in FoF and mobility self-efficacy is not yet established. In addition to its role in motor control, ACh may play an exacerbating role in the development of FoF. A better understanding of cholinergic changes throughout the brain may provide avenues to discover modifying therapies to improve the quality of life for those with PD. Therefore, we present this paper to examine the relationship between altered brain cholinergic activity and mobility self-efficacy in PD.

## 2. Materials and Methods

This study involved 126 subjects with Parkinson’s disease (95 males; 31 females), mean age 67.3 ± 7.1 (standard deviation (SD)) years. Participants were recruited from academic movement disorder clinics at the University of Michigan and Ann Arbor VA Healthcare system during 2015–2023. All subjects met the UK Parkinson’s Disease Society Brain Bank clinical diagnostic criteria [20]. Mean duration of disease was 6.4 ± 4.7 years. Subjects completed motor testing in the dopaminergic medication ‘off’ state. Mean modified Movement Disorder Society-Unified Parkinson’s Disease Rating Scale (MDS-UPDRS) motor score was 35.1 ± 8.3 [21], and modified Hoehn and Yahr disease severity ranged from stage 1.0 to 4.5 with a median stage of 2.5 [22]. Subjects completed the Montreal Cognitive Assessment (MoCA) with a mean score of 26.2 ± 3.1 [23]. 33 Subjects were taking a combination of dopamine agonist and carbidopa-levodopa medications, 75 were using carbidopa-levodopa alone, 6 were taking dopamine agonists alone, and 12 were not taking dopaminergic drugs. No subjects were treated with anti-cholinergic or cholinesterase inhibitor drugs.

Participants completed the 7-item short Falls Efficacy Scale (sFES-I) as a survey measure of FoF [8]. The sFES-I assesses a participant’s level of concern about falling while performing seven activities: dressing, bathing, getting in or out of a chair, going up or down stairs, reaching, going up or down a slope, and going out. Scores for each domain range from 1—Not at all concerned, to 4—Very concerned. Total scores are interpreted clinically in groups: low concern (7–8), moderate concern (9–13), and high concern (14–28).

This study (ClinicalTrials.gov Identifier: NCT02458430 and NCT05459753) was approved by the Institutional Review Boards of the Ann Arbor Department of Veterans Affairs Medical Center (protocol codes 1597500 & 1597134) and the University of Michigan (HUM00093414, HUM00197590, and HUM00168331). The affiliation Neurology Service and GRECC, VA Ann Arbor Healthcare System (VAAAHS) is part of the Ann Arbor Department of Veteran’s Affairs Medical Center. Written informed consent was obtained from all patients or their families/legal guardians.

### 2.1 Imaging

All subjects underwent magnetic resonance imaging (MRI) and vesicular acetylcholine transporter (VAChT) [^18^F]fluoroethoxybenzovesamicol ([^18^F]FEOBV) positron emission tomography (PET) imaging in a dopaminergic medication ‘on’ state per protocol. As the VAChT is the presynaptic molecular target of [^18^F]FEOBV PET imaging, we do not expect a significant effect of dopaminergic medication state to impact our results. MRI was performed on a 3T Philips Achieva system (Serial #42001, Philips, Best, The Netherlands) at the University of Michigan. PET imaging was performed in 3D imaging mode with a Siemens ECAT Exact HR+ tomograph or Biograph 6 Tru-Point PET/computed tomography (CT) scanner (Siemens Molecular Imaging, Inc., Knoxville, TN, USA), which acquires 63 transaxial slices (slice thickness: 2.4 mm) over a 15.2 cm axial field-of-view. Images were corrected for scatter and motion. [^18^F]FEOBV were prepared as described previously [24,25]. Delayed dynamic imaging was performed in frames of 5 minutes each for 30 minutes, 3 hours after an intravenous bolus dose injection of 8 mCi [^18^F]FEOBV.

Parametric images reflecting Distribution Volume Ratios (DVR) of [^18^F]FEOBV were constructed using the supratentorial white matter as a reference region as previously described [26–28]. The diameter of the sphere for erosion was 1mm. Structural MRI images were segmented into native and ‘Dartel-imported’ gray matter; white matter and cerebrospinal fluid using the Statistical Parametric Mapping 12 (SPM12) software package (https://www.fil.ion.ucl.ac.uk/spm/). The Müller-Gärtner method was used to reduce the partial volume effect (PVE) on the PET images [29]. The PVE-corrected PET images were co-registered with their corresponding structural MRI then normalized to the study specific template in Montreal Neurological Institute (MNI) space using high-dimensional DARTEL registration. The normalized, PVE-corrected PET images were spatially smoothed to 8 mm full width at half maximum (FWHM) to enhance the signal-to-noise ratio.

### 2.2 Statistical Analysis

A voxel-based correlation analysis of the parametric images in MNI space was performed using SPM12 to investigate the association between [^18^F]FEOBV binding and sFES-I scores. Whole brain analysis was conducted using Threshold-Free Cluster Enhancement (TFCE) to identify regions exhibiting significant relationships. TFCE results were corrected for multiple comparisons using family-wise error rate (FWE) correction (*p <* 0.05). Clusters that demonstrated significant negative correlations after FWE correction were considered statistically significant.

Mean DVR of all voxels from statistically significant clusters was extracted for all PD participants included in the analysis and additionally for a sample of 31 control subjects (15 males; 16 females), mean age 70.9 ± 7.2 (SD) years. To make the mean significant cluster DVR values interpretable with reference to control subjects, z-scores adjusted for effect of normal aging and biological sex were computed. A linear model was fitted predicting the mean DVR value from age and sex of controls, and subsequently used to predict the mean DVR value among patients. The difference between predicted and observed mean DVR value (residuals) among patients was calculated and divided by the standard deviation of residuals observed among control patients to yield the age and sex adjusted normative z-scores for the mean DVR of significant clusters from the SPM voxel-wise analysis (Z_*DVR*_). Z_*DVR*_ scores represent the deviation of patient [^18^F]FEOBV PET binding values from what would be expected if they were controls of equivalent age and sex, thereby offering a clearer reference frame for the outcome measure used in our post-hoc regression analyses. A post-hoc linear model was fitted predicting Z_*DVR*_ from sFES-I total scores and compared against the null intercept model using analysis of variance (ANOVA) F-test, with *α* significance threshold for the model comparison set at 0.05. Subsequently, a set of multivariate confounder regression models were fitted in a hierarchical fashion, including the additional effects of fall history (yes or no) and postural instability (having to take multiple steps or falling on the pull-back test) to assess whether the correlation between cholinergic system integrity and balance self-efficacy (our effect of interest) was independent of these measures of balance ability. Motor disease severity (MDS-UPDRS Part III total score), levodopa equivalent dose (LED), and years from symptom onset were added to assess whether our effect of interest was independent of overall disease progression. Cognitive efficacy as assessed by total MoCA score was added into the model due to previously reported on association between cholinergic integrity and cognition in PD [30]. Lastly, PD-specific effects of age and sex on cholinergic integrity (which would not have been covaried out by our z-scoring procedure) were added to the model to identify whether these demographic features may have a substantial bearing on our primary outcome parameter.

Regressors corresponding to each of the mentioned confounder variables were added individually into the model, and their influence on model goodness-of-fit was assessed using ANOVA F-test (*α* = 0.05) and adjusted coefficient of determination (*R*^2^). The influence of confounder regressors on our primary effect of interest was evaluated by examining the estimated standardized regression coefficients (*β*). Confounder regressors were retained in the model if their addition substantially improved model fit, modified *β* estimates of regressors already present in the model, or a theory-motivated reason was explicitly given for their retention. Confounder regressors not meeting any of the conditions for retention were excluded from subsequent confounder regression steps. A set of diagnostics were performed on the regression model of best fit obtained from the described procedure. Variance inflation factor (VIF) corresponding to each of the regressors was examined to identify any excessive multicollinearity. Distribution of model residuals was examined to ensure the normality of model residuals, and a robust linear model of same specification was fitted in case evidence for non-normality of residuals was observed.

## 3. Results

**Fig. 1.**
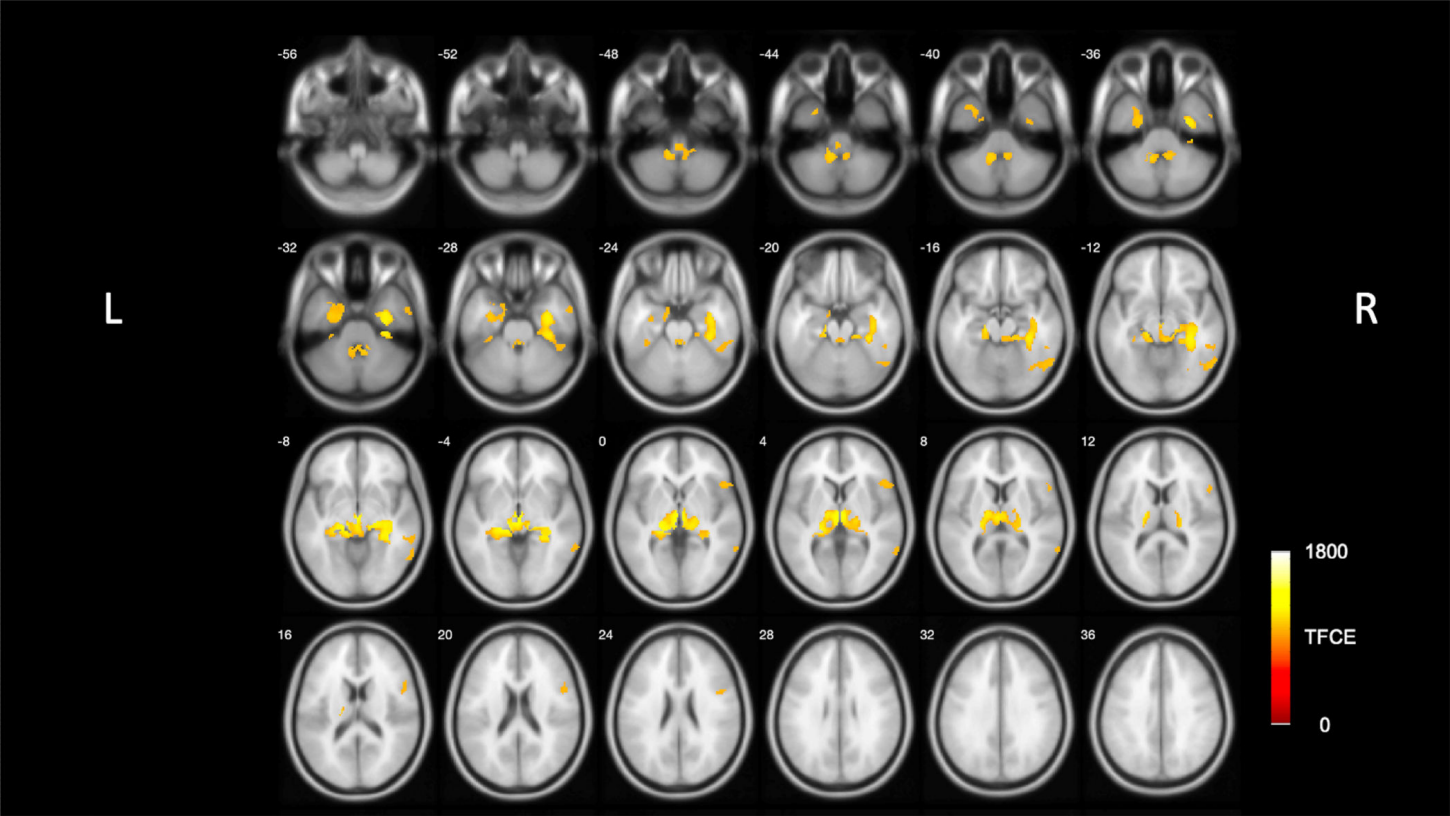
Voxel-based vesicular acetylcholine transporter positron emission tomography (PET) and total short Falls Efficacy Scale (sFES-I) score analysis. Significant clusters of the negative correlation between total sFES-I scores and regional vesicular acetylcholine transporter (VAChT) PET binding as shown in Threshold-Free Cluster Enhancement (TFCE)-scores superimposed on brain magnetic resonance imaging (MRI) atlas images family-wise error rate-corrected *p <* 0.05. Significant findings (shown in neurological orientation (L = left, R = right)) are localized to the tectum, metathalamus (LGN more than MGN), thalamus, hippocampus, parahippocampus, fusiform gyri, and fimbriae. PET imaging findings superimposed on International Consortium for Brain Mapping (ICBM) adopted Montreal Neurological Institute (MNI) MRI T_1_-weighted template. LGN, lateral geniculate nucleus; MGN, medial geniculate nucleus.

Whole brain voxel analysis using TFCE showed significant negative, FWE-corrected (*p <* 0.05), correlations between total sFES-I scores and VAChT expression. Five peak cluster locations were identified, including the left fusiform gyrus (2 clusters), left mediodorsal thalamus, right middle temporal gyrus, and right inferior frontal gyrus. Significant correlations between cholinergic denervation and sFES-I scores were noted in the tectum, lateral geniculate nucleus (LGN), medial geniculate nucleus (MGN), thalamus proper, mesiotemporal lobe, and right cerebellum lobules III and VI (Fig. 1, Table 1). No positive correlations were identified.

**Fig. 2.**
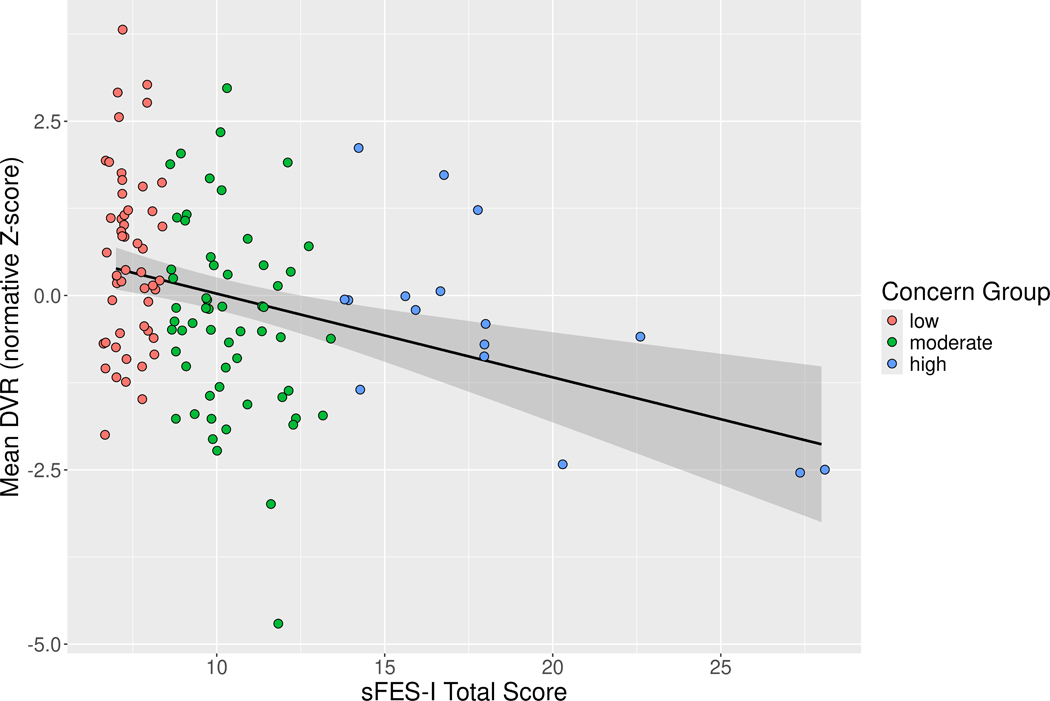
Scatter plot of sFES-I scores by mean [^18^F]FEOBV DVR values. Subject sFES-I total scores were plotted in relationship to their mean [^18^F]FEOBV DVR values from significant clusters. Subject scores were color coded to show whether they fell into the low concern group (n = 52, red), moderate concern group (n = 58, green), or high concern group (n = 16, blue). Slight jitter was added in x-axis to avoid excessive overlaps of scatter points. DVR, Distribution Volume Ratios.

The normative z-scoring model fitted on a sample of 31 control subjects yielded a significant effect of biological sex among controls, with female control participants tending to have higher [^18^F]FEOBV uptake in regions found to associate with sFES-I scores in PD subjects (*β* = 0.13 [0.04, 0.21], *p* = 0.004), and no statistically significant effect of age among controls (*β* = 0.00 [0.0, 0.01], *p* = 0.664), with the total model accounting for a fifth of the variance in examined regional binding among control participants (*R*^2^ = 0.209, *F* = 4.958, *p* = 0.014). Fig. 2 depicts computed mean Z_*DVR*_ scores plotted against sFES-I total scores among our PD subjects.

The post hoc analysis demonstrated that sFES-I total scores account for a significant proportion of variance in [^18^F]FEOBV uptake of regions found to be significant in the voxelwise analysis (*R*^2^ = 0.101, *F* = 15.01, *p <* 0.001), with higher sFES-I scores associated with lower uptake (*β* = −0.46 [−0.69, −0.23], *p <* 0.001). In the subsequent confounder analysis neither model fit nor sFES-I coefficient estimate were significantly impacted by falls history (*F* = 0.422, *p* = 0.52), MDS-UPDRS-III (*F* = 0.0854, *p* = 0.77), LED (*F* = 1.346, *p* = 0.25), disease duration (*F* = 1.411, *p* = 0.49), or sex in the PD sample regressors (*F* = 1.411, *p* = 0.24) at the point of their addition to the model (see Supplementary Material Section 1 for detailed step-by-step model building process). The final model of best fit accounted for approximately 40% of the variance in [^18^F]FEOBV uptake (*R*^2^ = 0.393). The effect of sFES-I total scores on [^18^F]FEOBV uptake remained statistically significant in the model of best fit (*β* = −0.3 [−0.5, −0.1], *p* = 0.004), after controlling for effects of postural instability, MoCA total score, and age in PD sample as measure of disease progression (see Table 2 for model coefficients). Model diagnostics indicated no evidence of substantial multicollinearity in the model of best fit but some evidence of non-normality in model residuals which were found to be left-skewed (skewness = −0.65) and leptokurtic (kurtosis = 7.52). An alternative robust linear model of the same specification as the original linear model was fitted, and the regression coefficient for effect of sFES-I total scores remained statistically significant (*β* = −0.26 [−0.45, −0.08], *p* = 0.006), suggesting that non-normality of model residuals in our original model of best fit did not substantially affect the estimate for the regression coefficient of primary interest to the present work (see Supplementary Material Section 2 and Section 3 for details).

## 4. Discussion

Our findings indicate that reduced ACh availability in the tectum, metathalamic nuclei (LGN and MGN), thalamus, mesiotemporal lobe, and right cerebellum (lobules III and VI) correlated with greater FoF. Furthermore, higher sFES-I scores associated with lower [^18^F]FEOBV binding independent of other confounders (disease progression, cognition, and objective postural instability) and remained a significant predictor of VAChT binding. This supports our hypothesis that FoF in PD may be influenced by the degradation of cholinergic system integrity. Our results point to large scale network dysfunction of the cholinergic basal forebrain and brainstem. The medial septal nucleus (Ch1) and the vertical limb of the diagonal band (Ch2) provide cholinergic innervation to the hippocampus while the nucleus basalis of Meynert (Ch4) innervates the temporal lobe including the parahippocamus. Furthermore, our results implicate cholinergic brainstem nuclei projections, including PPN-LDTC (Ch5 and Ch6) projections to the thalamus and other forebrain structures, parabigeminal nucleus projections to the colliculus, and cholinergic medial vestibular neurons projections to the cerebellum. Many of these regions that we have identified as having reduced cholinergic activity are associated with sensory processing roles.

Within the mesiotemporal lobe, the hippocampus, parahippocampus, fusiform gyri and fimbriae showed lower cholinergic activity. These areas are known to play a key role in many cognitive tasks such as memory formation, spatial navigation, and emotional processing. The fusiform gyrus, also known as the occipitotemporal gyrus, is thought to play an important role in visual categorization, specifically in high spatial frequencies processing required for tasks such as reading, face perception [31], and object recognition [32]. The recognition and interpretation of high spatial frequencies, which provides detailed information regarding a stimulus, is one part of the puzzle that is necessary for accurate scene perception [33].

Additionally, the hippocampus plays a large role in how we navigate and experience our world through episodic memory, the memory of experiences set in a spatial-temporal context, and semantic memory, the stored general or factual knowledge of the world. The cognitive map theory asserts that the hippocampus is necessary for allocentric spatial representations of the environment which offers context for episodic events [34,35]. The hippocampus is thought to be responsible for the retrieval of these spatial representations as cognitive maps. Furthermore, using virtual simulation and functional Magnetic Resonance Imaging (fMRI), Javadi *et al.* [36] demonstrated that posterior hippocampus activity increased in response to a greater number of paths whereas anterior hippocampus activity corresponded with global properties. This relates to the ability to catalogue route information necessary for path finding and planning future routes. Furthermore, the multiple trace theory states that the hippocampus is vital for recreating mental experiences of all spatial and episodic memories, regardless of when they occurred, which then helps to develop schematic spatial maps and semantic memories [35]. This theory suggests semantic-like spatial memory plays a role in cognitive mapping as it allows for a familiar location to evoke spatial navigation skills without fully re-experiencing previous memories. This may be applicable to utilizing spatial generalizations when navigating a new area. Dysfunction in this area may increase uncertainty when planning a route, making navigation more difficult, which could potentially increase FoF in those with PD, especially in novel locations.

The parahippocampal cortex (PHC) is seated between the fusiform gyrus and the hippocampus. Within the PHC is an area known as the parahippocampal place area (PPA) which responds to stimuli depicting scenes and enables the encoding of the local environment [37]. This area has been shown to respond selectively to scenes in comparison to single objects. It has been posited that the PPA plays a crucial role in selecting the correct cognitive map to allow for navigation [38]. This was elaborated on by Sun *et al*. [39] who demonstrated the significance of the right PPA in encoding the spatial associations of significant landmark objects for indoor locations. Another recent fMRI study showed that the PHC is active when completing a distance estimation task with a stable landmark [40]. The same level of PHC activation was not noted when the landmark was ambiguous. This fits with the theory of Aminoff *et al*. [41] which suggests that although the PHC plays a role in many different processes such as spatial and episodic processing, its role is truly to provide contextual associations between different stimuli, in this case a spatial association between the goal destination and landmark. This implies that the PHC is a vital component of the environmental processing necessary for navigation. Impaired ability to form appropriate contextual associations, including spatial associations necessary to navigate our environment, may be associated with greater FoF in PD.

Research suggests the PHC also plays a role in integrating visual and emotional stimuli. The PHC, specifically the right PPA, has been shown to respond more to threatening background scenes than non-threatening scenes [42]. The left PHC displays similar differential responsiveness to auditory stimuli (*e.g*., differential response to happy *vs.* neutral music) [43]. Cholinergic changes in these areas may disrupt emotional processing, altering the categorization of threatening *vs.* non-threatening scenes. Such emotional processing disruptions may alter how PD patients react to their environment, inflating fear response to perceived dangers and lowering confidence in their ability to navigate safely.

The relationship between cholinergic deficits in the thalamus and sFES-I scores supports that sensory dysfunction may play a role in increasing FoF in PD. The thalamus plays an important role in relaying sensory and motor information. Previous researches have identified the thalamus as a contributor to the integration of posture-related sensory input [44–46]. In particular, cholinergic changes in the LGN, which processes visual information, have been implicated in increased motor impairments in PD including falls and freezing of gait [47]. The thalamus and temporal limbic system are interconnected, sending signals bi-directionally that are important for sensory processing, learning, and memory. Our findings suggest that the brain’s ability to integrate visuomotor input may be impaired in PD, and that impairment may contribute to an increased fear of falling.

Furthermore, deficits in cholinergic network integrity have been implicated in vestibular dysfunction. The MGN is affected by the vestibulocochlear nerve, consisting of the vestibular and auditory nerves. Prior work by our group showed cholinergic deficits in these areas are linked with vestibular dysfunction and PIGD features in PD [48]. While the MGN is considered part of the auditory thalamus, it also receives visual, somatosensory, and nociceptive inputs to its medial subdivision [49]. Vestibular dysfunction, compounded by impaired sensory processing may increase balance and gait difficulties and perpetuate fear of falling. Other recent work with blood oxygen level-dependent (BOLD) activity has shown that there is bilateral thalamic activity in the left medio-dorsal and right anterior thalamus for touch, mechanical pain, and vestibular stimulation [50]. Sensory integration deficits across sensory modalities may play a large role in developing FoF. While past research has implicated thalamo-temporal-limbic cholinergic changes with physical contributors to fall risk, the present findings suggest that these changes associate with neurobehavioral risk factors as well.

The tectum, consisting of the superior and inferior colliculi, is involved in attention, sensory processing, and head and eye movements. Specifically, the superior colliculus plays a key role in spatial attention, including the target selection that precedes movements [51]. Within PD, the tectal area is implicated in abnormal saccades, the rapid eye movements between fixation points [52]. Saccade abnormalities, including increased latency and variability have been associated with freezing of gait in PD [53]. PD patients with freezing of gait have also been documented to fixate more on proximal areas than those without freezing of gait and healthy controls [54]. The superior colliculus has been implicated as an attention-orientator [55]. Researches have demonstrated that in many animals it enables looming-collision detection behavior [56,57]. In rodents this has been noted to result in freezing of movement [58]. Emotional dysregulation may also have a negative effect on tectal control of eye movements which could potentially inhibit safe or confident ambulation. The superior colliculus receives cholinergic inputs from the pedunculopontine tegmental nucleus and parabigeminal nucleus. Reduced cholinergic activity in the superior colliculus may play a role in freezing of gait when encountering a situation that is associated with FoF. In addition, the superior colliculi may affect the basal ganglia through the intralaminar nuclei of the thalamus which creates a circuit involving the caudate and putamen, substantia nigra pars reticulata, and back to the superior colliculus. Losses of cholinergic nerve terminals in the superior colliculus may also result in dysfunction of multiple non-motor circuits. The superior colliculus may contribute to pathways that are routed through the thalamus involved in response to visual stimuli and attention tasks through downstream modulation of the cortex [51]. These areas are impacted by the progression of PD and impaired function of this area may compound both motor and non-motor symptoms.

Our findings indicate that areas of the cerebellum have decreased cholinergic integrity associated with FoF. The cerebellum is a massive network of neurons and is involved with spatiotemporal firing patterns [59]. Cerebellar lobe VI is included in circuitry involving sensorimotor and spatial processing [60]. This structure is also vital for procedural memory, which facilitates learned, automatic activities like riding a bike [61]. Disruptions of this circuitry may increase the difficulty of doing everyday tasks and contribute to a FoF. The cerebellum also impacts processes of fear learning and extinction through projections to the limbic system, thalamus, and dopaminergic neurons [62]. The effect of cholinergic losses may be further felt through the emotional dysregulation related to cerebellar function.

A limitation of this study is the predominance of male subjects, limiting the generalizability of our findings to a larger, more diverse Parkinson’s disease population. Additionally, as the analysis is cross-sectional in design, the conclusions that can be made about relationships found in this study and how they may change with disease progression are limited. More research is needed to better understand the relationship between cholinergic changes and fear of falling in PD. While our findings indicate that vital regions responsible for spatial contextualization like the hippocampus, thalamus and parahippocampal cortex are significant contributors to FoF in PD, other regions may prove to be of interest for future research to fully understand mobility self-efficacy and FoF in this population.

## 5. Conclusions

In this study, we found that cholinergic denervation in key sensory processing networks associated with fear of falling in PD. Our findings parallel past research that implicates cholinergic deficits in regions of the thalamus and hippocampus with postural instability and gait disturbances in PD. The involvement of the parahippocampal cortex in addition to the tectum, LGN, and hippocampus as shown in the present findings suggests that impairment to the brain’s ability to integrate visual stimulus and motor response could drive down balance confidence, increasing fear of falling in PD.

As fear of falling represents a risk factor for actual falls independent of motor impairment, a better understanding of the neurobiology underlying fear of falling, and how it interfaces with motor impairments could inform better fall-prevention strategies and interventions. For example, physicians could employ cognitive behavioral therapy to help patients understand that their balancing ability may be stronger than their fear response implies. Physical therapies could focus on specific exercises to build strength and confidence in areas that trigger FoF. An integrated approach to treatment that encourages physical activity and mindfulness could reduce sedentariness, frailty, and mitigate fall risk, ultimately increasing overall quality of life in PD.

## Supplementary Material

Supplementary files

## Figures and Tables

**Table 1.** Significant sFES-I-associated [^18^F]FEOBV binding clusters.

Cluster (voxels)	Peak MNI Coordinates			TFCE value	Brodmann Area	Peak Voxel location	Predominant Network Hub
	X	Y	Z				
535	−32	−8	−30	1047	28, 34, 35, 36, 38	Left fusiform gyrus	Left fusiform gyrus Left parahippocampal Left hippocampus Left inf-mid temporal pole

4726	−4	−16	2	1249	19, 20, 21, 22, 27, 28, 30, 35, 36, 37	Left mediodorsal thalamus	Bilateral hippocampus Bilateral parahippocampal Bilateral lingual. Bilateral mediodorsal thalamus Bilateral ventral thalamus Bilateral intralaminar thalamus Bilateral metathalamus Left IV, V, IX lobule of cerebellar hemisphere Right fusiform gyrus Right amygdala Right III, IV, V VI and IX lobule of cerebellar hemisphere Midbrain Vermis III and X

75	58	0	−28	996	20, 21	Right mid temporal gyrus	Right mid temporal gyrus Right Inf temporal gyrus

54	−34	−34	−27	975	20	Left fusiform gyrus	Left fusiform Left IV, V, VI lobule of cerebellar hemisphere

220	52	22	2	1012	44, 45, 47	Right inferior frontal gyrus	Right inferior frontal gyrus

Significant sFES-associated [^18^F]FEOBV binding clusters with a minimum of 50 voxels with the location of the peak voxel, peak voxel TFCE-score, and the regions associated with the clusters, *p <* 0.05, FWE correction after Threshold free cluster enhancement (SPM). [^18^F]FEOBV, [^18^F]fluoroethoxybenzovesamicol; FWE, family-wise error; MNI, Montreal Neurological Institute; TFCE, Threshold-Free Cluster Enhancement; SPM, Statistical Parametric Mapping; sFES, Short Falls Efficacy Scale.

**Table 2.** Final model of best fit.

Predictors	[^18^F]FEOBV Z_*DVR*_		
	*β*	95% CI	*p*
(Intercept)	0.18	−0.15 to 0.51	0.284
sFES-I total score	−0.30	−0.50 to −0.10	**0.004**
Postural instability	−0.27	−0.70 to 0.16	0.217
MoCA total score	0.21	0.01 to 0.41	**0.042**
Age	−0.66	−0.86 to −0.45	*<* **0.001**
Observations	126		
*R*^2^/*R*^2^ adjusted	0.413/0.393		

The final model of best fit includes sFES-I total, postural instability, MoCA, and age. Postural instability was no longer significant when factoring in other confounders but was retained in the model to demonstrate that the effect of sFES-I is independent of objective postural control impairment. This model accounts for approximately 40% of the variance in [^18^F]FEOBV uptake. sFES-I remains a significant predictor of [^18^F]FEOBV cluster binding. MoCA, Montreal Cognitive Assessment; CI, confidence interval. Significant *p*-values are bolded.

## Data Availability

The data are not publicly available because they contain information that could compromise the privacy of the research participants. The data that support the findings of this study are available on reasonable request from the corresponding author.
