# Supplementary material for: Topography of Cholinergic Nerve Terminal Vulnerability and Balance Self-Efficacy in Parkinson’s Disease
Source: J Integr Neurosci. Author manuscript; Available in PMC 2025 May 17. (PMC12084976; doi:10.31083/j.jin2309178)
Supplement: Supplementary files [file NIHMS2057542-supplement-Supplementary_files.docx]

**Supplementary Materials**

*Section 1. Detailed Regression Modelling*

The model with sole inclusion of the sFES total score regressor demonstrated as statistically significant negative correlation, wherein higher scores were associated with lower [^18^F]FEOBV uptake (*β*=-0.46 [-0.69, -0.23], *p*<0.001) (Table 1).

Table 1. sFES association with lower [^18^F]FEOBV uptake


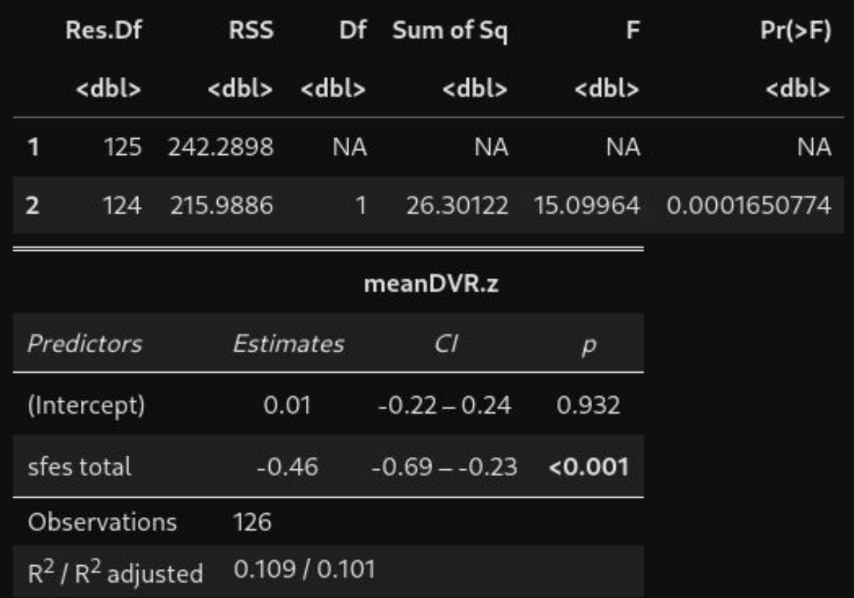


The regression model with sFES scores and lower [^18^F]FEOBV uptake shows a significant negative correlation

The addition of fall history to the model did not substantially improve model fit (*F*=0.422, *p*=0.517)*,* and the regression coefficient for sFES total score remained essentially unchanged (*β*=-0.44 [-0.68, -0.2], *p*<0.001), suggesting that fear of falls contributes to [^18^F]FEOBV uptake independently of history of falls (Table 2).

Table 2. Regression model with addition of fall history


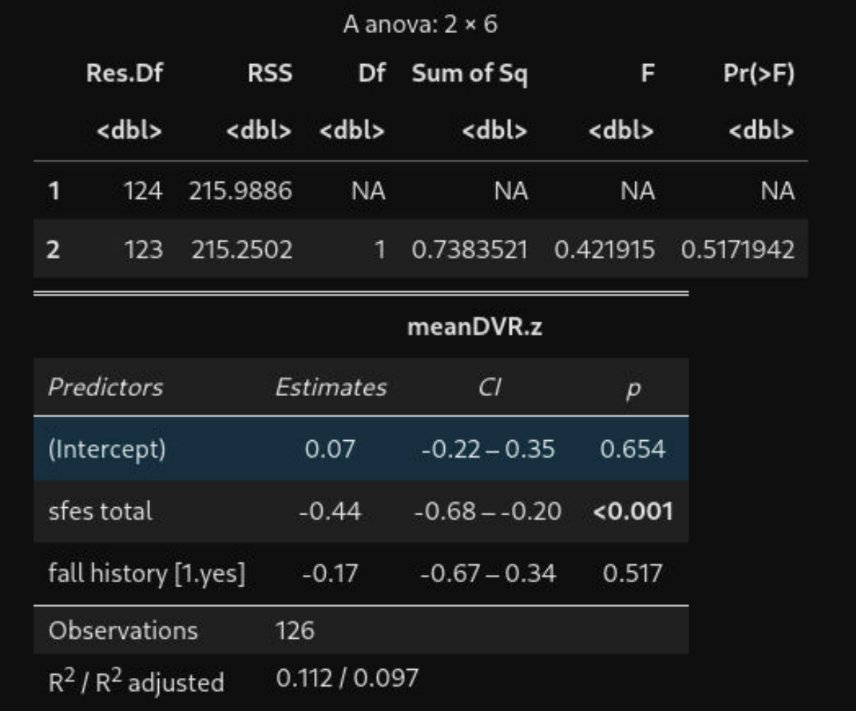


The addition of fall history as a variable did not significantly impact the regression model.

The addition of postural instability to the model substantially improved model fit (R^2^=0.159, *F*=9.513, *p*=0.00252)*,* with presence of postural instability associated with lower [^18^F]FEOBV uptake (*β*=-0.75 [-1.23, -0.27], *p=*0.003). The regression coefficient for sFES total score was slightly weakened by the addition of postural instability to the model but remained statistically significant (*β*=-0.38 [-0.61, -0.15], *p=*0.002), suggesting that some portion of cholinergic losses related to both imbalance and fear of falls is shared, with fear of falls nevertheless contributing independently to cholinergic losses. Interestingly, the addition of postural instability regressor also made the intercept term significantly higher than 0 (mean of age & sex-equivalent controls), suggesting that PD patients with average sFES scores in our sample and no postural instability tend to have slightly higher FEOBV uptake than controls. See table 3 for results.

Table 3. Regression model with addition of postural instability


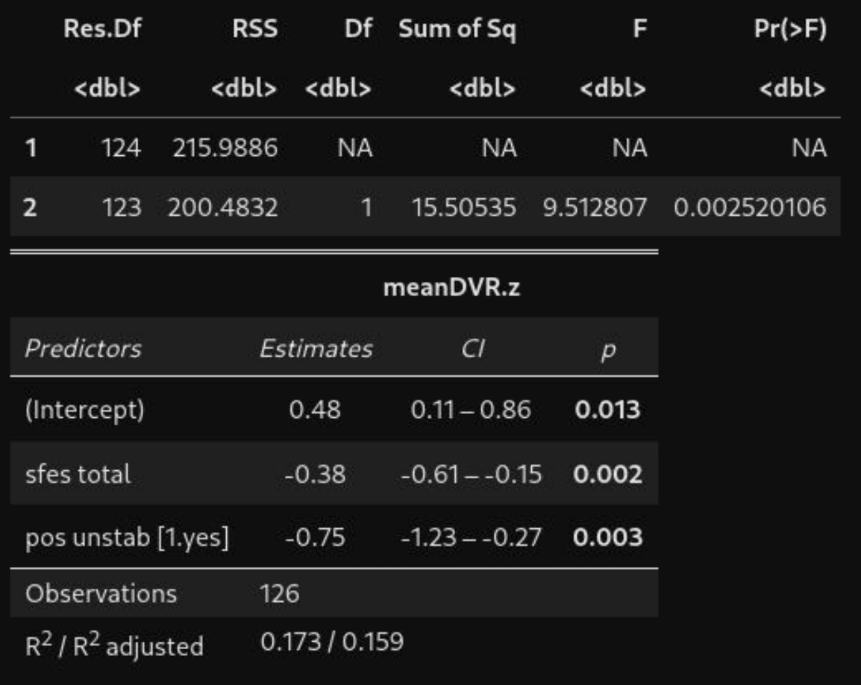


Postural instability significantly impacted the model fit (R^2^=0.159, *F*=9.513, *p*=0.00252)*,* with presence of postural instability associated with lower [^18^F]FEOBV uptake (*β*=-0.75 [-1.23, -0.27], *p=*0.003).

The addition of Movement Disorder Society-Unified Parkinson’s Disease Rating Scale (MDS-UPDRS part III) total scores did not substantially improve model fit and did not affect the estimate for either regression coefficient presently in the model. This suggests that specifically advanced postural instability related symptoms and not overall disease severity associates with cholinergic losses in overlapping regions to those which correlate with fear of falls.

Table 4. Regression model with addition of MDS-UPDRS part III


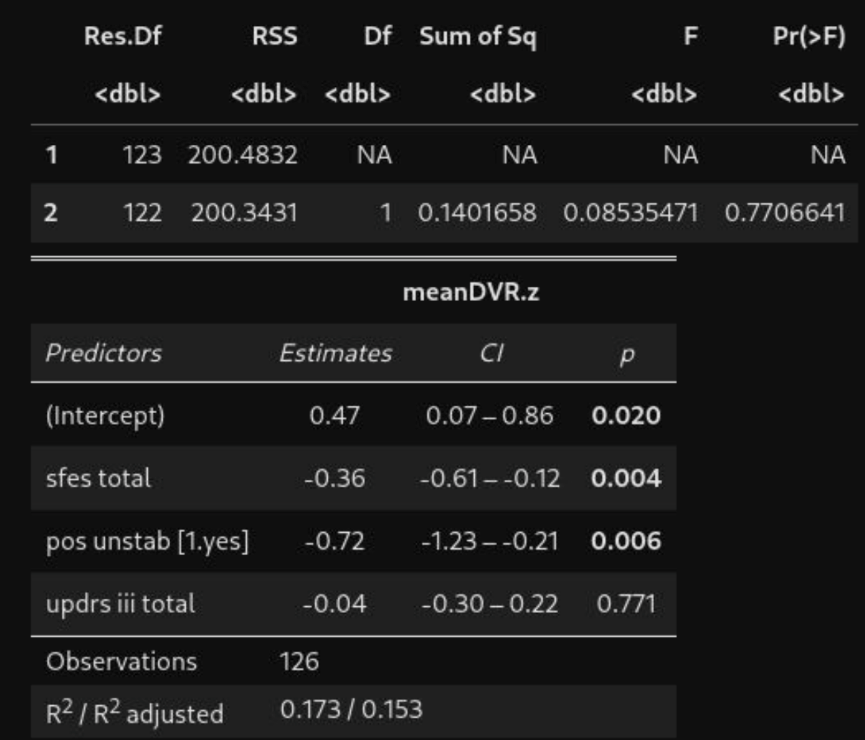


The addition of MDS-UPDRS part III did not significantly change the model.

Neither the addition of levodopa equivalent dose (Table 5) nor disease duration (years from symptom onset) (Table 6) substantially improved model fit nor affect the estimates for regression coefficients already in the model, further supporting the specificity of cholinergic integrity in examined regions to postural stability and fear of falls related manifestations of Parkinson’s disease, and not to overall disease progression.

Table 5. Regression model with addition of levodopa equivalent dose (LED)


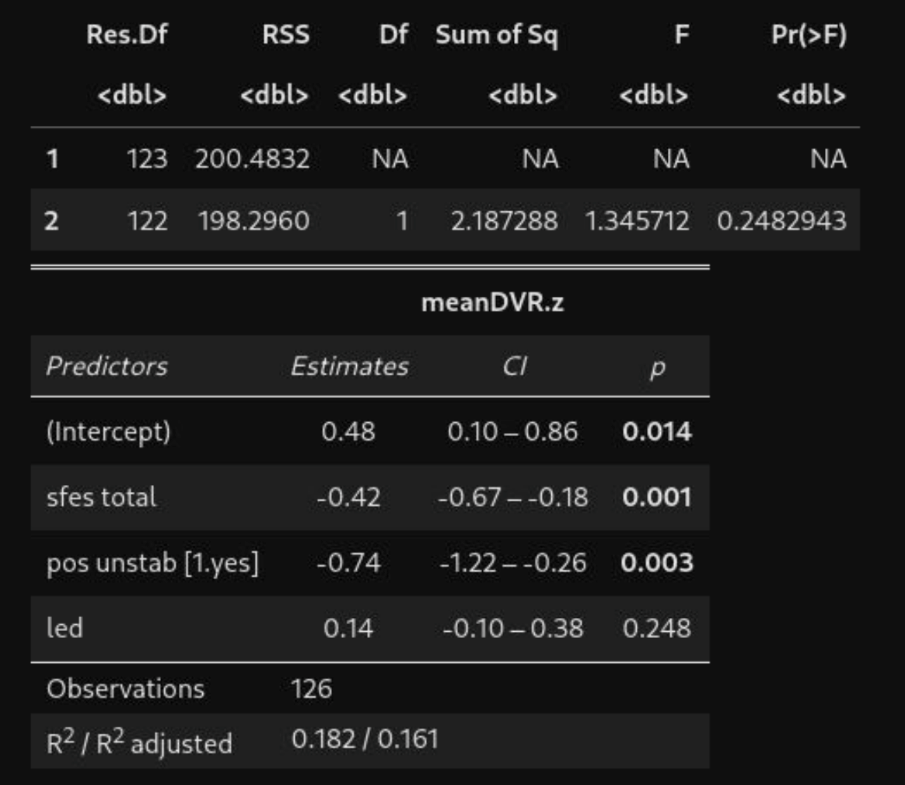


Table 6. Regression model with addition of disease duration


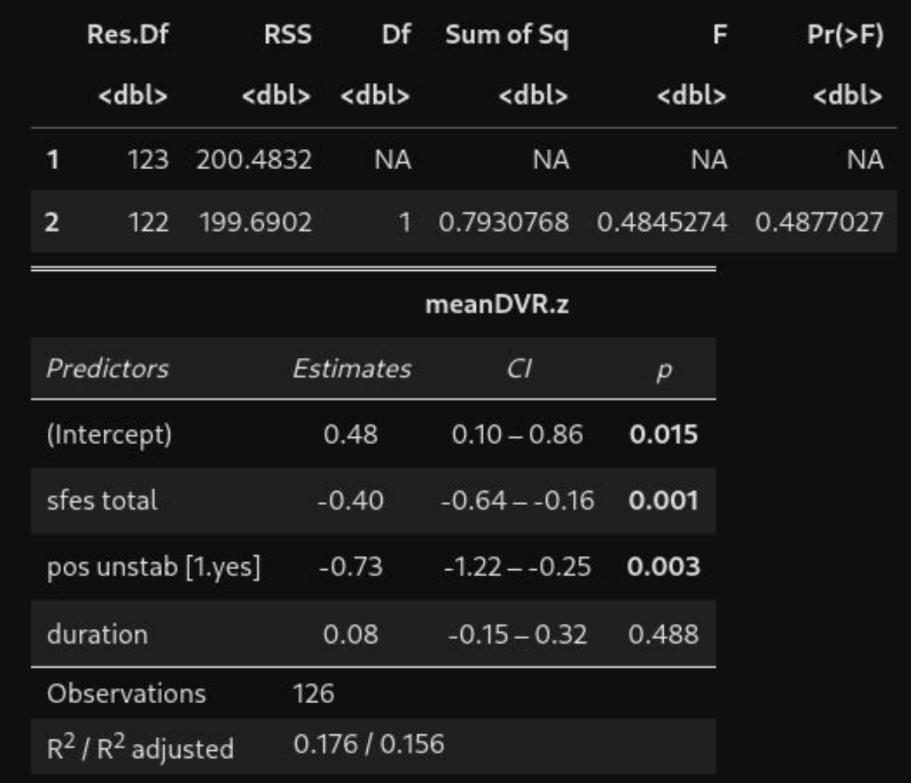


Neither LED or disease duration had a significant effect on the model, as shown in table 5 and table 6 respectively.

The addition of Montreal Cognitive Assessment (MoCA) scores as a measure of cognitive efficacy substantially improved model fit (*R*^2^=0.2, *F*=7.265, *p*=0.008), with higher cognitive efficacy associated with higher cholinergic integrity in examined regions (*β*=0.31 [0.08, 0.54], *p=*0.008) (Table 7). The estimates for both sFES total score (*β*=-0.31 [-0.54, -0.08], *p=*0.008) and postural instability (*β*=-0.67 [-1.14, -0.2], *p=*0.006) appeared to be slightly weakened by the addition of MoCA scores, but however remained statistically significant. This suggests that greater risk of falls associated with loss of cholinergic integrity in examined regions independently of overall cognitive decline.

Table 7. Regression model with addition of MoCA


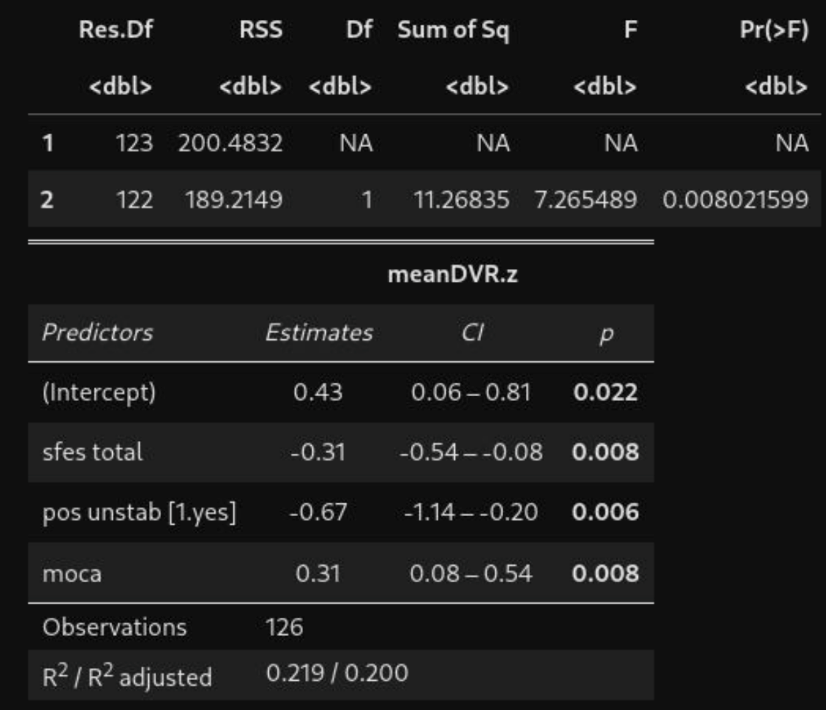


No significant effect was observed when MoCA was added into the model.

The addition of sex did not significantly improve model fit, or substantially affect the sFES total score regression coefficient (Table 8).

Table 8. Regression model with addition of sex


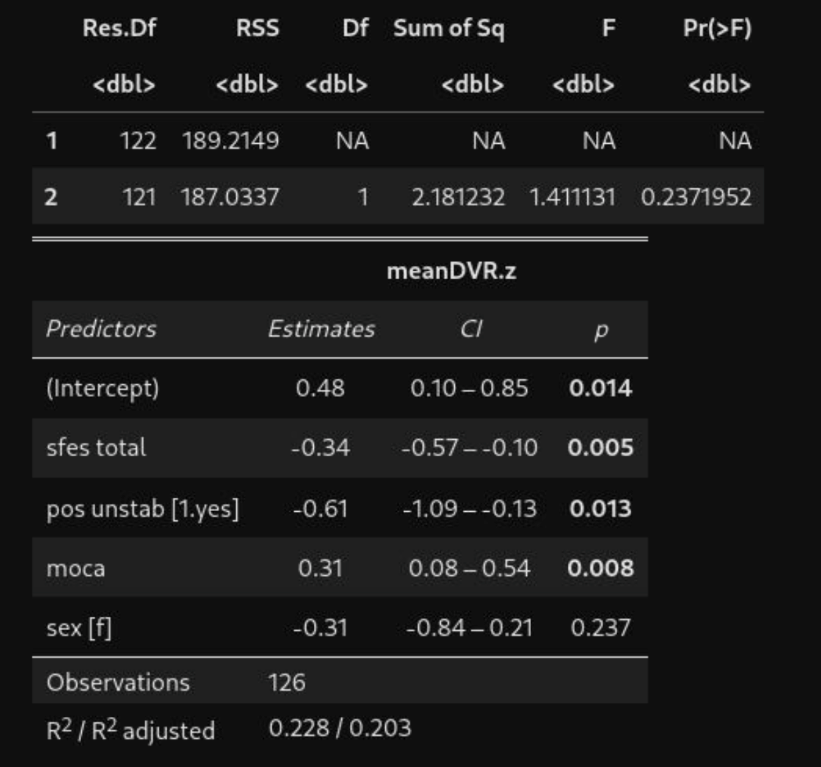


Sex did not have a significant effect on the model

The addition of age to the model substantially improved model fit (*R*^2^=0.393, *F*=39.93, *p<*0.001), with higher age associated with lower cholinergic integrity among examined regions in our sample of PD patients (*β*=-0.66 [-0.86, -0.45], *p<*0.001). The estimates for effect of sFES total score remained essentially unchanged, whereas the estimate for effect of postural instability was substantially weakened and was no longer statistically significant (*β*=-0.27 [-0.7, 0.16], *p=*0.217). See these results in table 9. Given that the Z_DVR_ values used as the dependent measure in our regression analyses already covary out the effect of age and sex as observed among control patients, we conjecture that the effect of age in the present model represents overall disease progression, a possibility that is supported by the fact that the variance explained by postural instability (a symptom of advanced PD) strongly overlapped with the variance explained by age. Independence of sFES total score regressor from age suggests that patients with greater fear of falls might represent a more malignant subpopulation of PD patients with greater cholinergic losses than would be expected at a given stage of objective postural impairment, overall disease progression, and cognitive efficacy. Interestingly, upon the addition of age, the intercept term was rendered no longer significantly greater than 0, suggesting that possible upregulation of [^18^F]FEOBV uptake among examined regions in PD patients is disease progression dependent, manifesting only in earlier stages of the disease.

Table 9. Regression model with addition of age


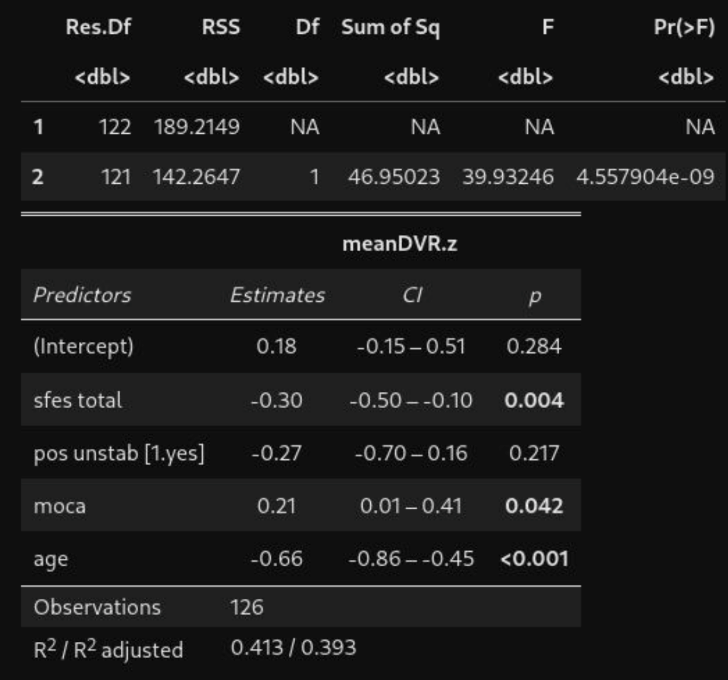


Age substantially improved model fit (*R*^2^=0.393, *F*=39.93, *p<*0.001). The estimate for effect of postural instability was substantially weakened and was no longer statistically significant (*β*=-0.27 [-0.7, 0.16], *p=*0.217).

*Section 2. Model diagnostics*

The variance inflation factor for all the regressors included in the model of best fit fell far below 2 (Table 10), suggesting absence of evidence for substantial multicollinearity.

Table 10. Variance inflation factor for significant regressors


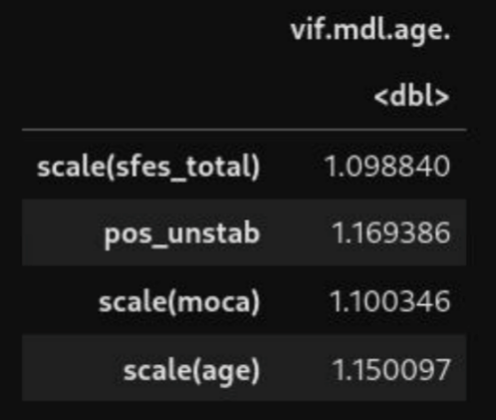


No variance inflation factor for any regressors surpassed 2 suggesting absence of evidence for substantial multicollinearity.

The distribution of final residuals for the model of best fit was plotted as a histogram and tested with Shapiro-Wilks test of normality (Figure 1). Both visual inspection and statistical test presented evidence for non-normality of model residuals. Based on visual inspection and skewness and kurtosis descriptive statistics it was identified that the distribution of residuals deviates from normality in that it is left-skewed and leptokurtic.


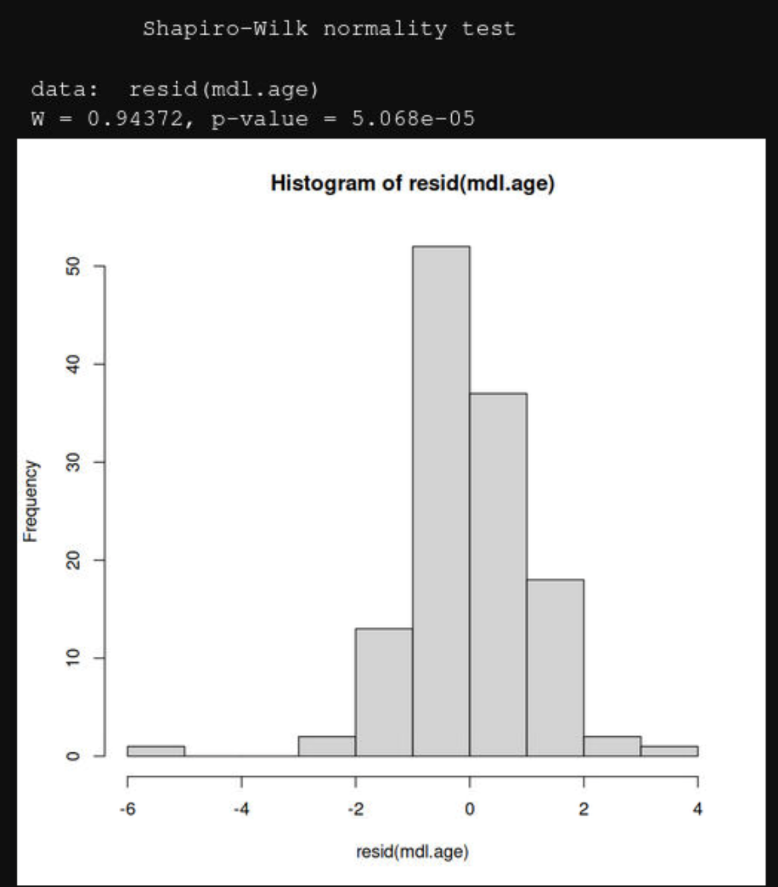


Fig 1. Histogram of distribution of final residuals. The distribution of final residuals for the model of best fit as a histogram and tested with Shapiro-Wilks test of normality. Distribution of residuals deviates from normality in that it is left-skewed and leptokurtic

We fitted a robust linear model with the same specification as our linear model of best fit and found that regression coefficient estimate for the effect of sFES total score remained statistically significant and still trended in the same direction (Table 11). The effect of age also remained statistically significant, however the effects of MoCA scores and postural instability lost their statistical significance. Given our main interest was in the effect of sFES total scores, we conclude that violation of normality assumption did not substantially bias the main conclusions of our present work.

Table 11. Results of robust linear modeling


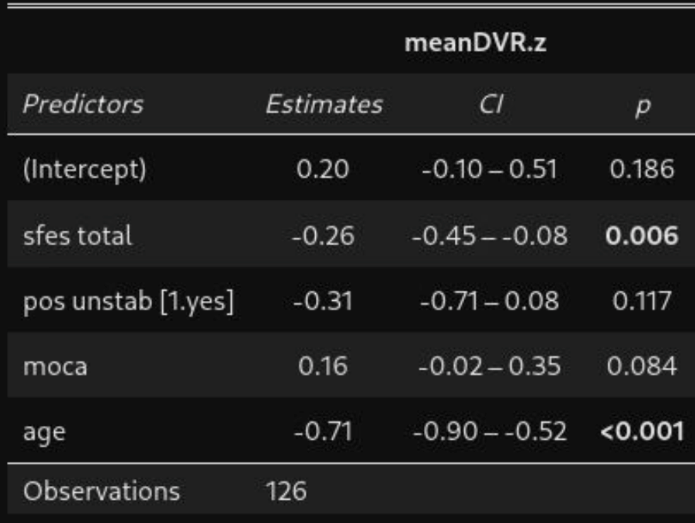


The regression coefficient estimate for the effect of sFES total score remained statistically significant and trending the same direction.

*Section 3. Multivariate Analysis by sFES-I Concern Group*

**Methods**

To better characterize the association between clinical concern level sFES subgrouping (low, moderate, and high concern), and relevant co-variates used in the post-hoc multivariate analysis, a set of ANOVA group comparisons were performed for continuous co-variates, and a set of chi-square contingency tests were performed for categorical variables.

**Results**

ANOVA group comparisons and chi-square contingency tests demonstrated that the moderate concern group appeared to exhibit a relatively isolated decrease in cholinergic integrity of examined regions and somewhat elevated proportion of postural unstable patients. The high concern group appeared to exhibit an equivalent extent of decrease in cholinergic integrity relative to the low concern group, but also had higher motor symptom severity, worse cognition, longer disease duration, higher levodopa equivalent dose, and even greater incidence of postural instability.

Table 12. ANOVA group comparisons and chi-square contingency tests for categorical variables

| *Variable \| Subgroup* | *Low Concern (Reference, N=52)* | *Moderate Concern (N=58)* | *High Concern (N=16)* | *Statistic* | *P-value* |
| --- | --- | --- | --- | --- | --- |
| sFES-I  (total score) | 7.385  [6.903, 7.866] | **+2.977***  **[2.314, 3.641]** | **+10.865***  **[9.872, 11.858]** | F=235.474 | **<0.001*** |
| Age  (years) | 66.077  [64.142, 68.011] | +2.389  [-0.276, +5.053] | +0.673  [-3.315, +4.661] | F=1.623 | 0.201 |
| Sex  (female/male, %female) | 12/40  (23.1%) | 18/40  (31%) | 1/15  (6.2%) | χ^2^=4.264 | 0.119 |
| MoCA  (total score) | 26.635  [25.784, 27.485] | -0.428  [-1.599, +0.743] | **-1.885***  **[-3.638, -0.131]** | F=2.264 | 0.108 |
| MDS-UPDRS part III  (total score) | 34.038  [30.405, 37.672] | +4.72  [-0.283, +9.724] | **+16.118***  **[+8.628, +23.608]** | F=9.148 | **<0.001*** |
| Disease duration  (years from onset) | 5.827  [4.563, 7.091] | +0.57  [-1.171, +2.31] | **+2.798***  **[+0.193, +5.403]** | F=2.266 | 0.108 |
| LED  (mg) | 581.577  [477.859, 685.295] | +5.311  [-137.525, 148.147] | **+306.236***  **[+92.415, +520.056]** | F=4.507 | **0.0129*** |
| Fall History  (yes/no, %yes) | 14/38  (26.9%) | 22/36  (37.9%) | 6/10  (37.5%) | χ^2^=1.638 | 0.441 |
| Postural instability  (yes/no, %yes) | 26/26  (50%) | 40/18  (69%) | 14/2  (87.5%) | χ^2^=8.812 | **0.0122*** |
| Mean [^18^F]FEOBV DVR (normative Z-score) | 0.529  [0.163, 0.895] | **-0.868***  **[-1.371, -0.364]** | **-0.941***  **[-1.695, -0.187]** | F=6.731 | **0.00168*** |

Significant differences between groups were found for postural instability, LED, MDS-UPDRS part III, sFES-I scores and Mean [18F]FEOBV DVR

*“*” indicates a significant difference.*
